# Advancing basic and preclinical spine research: Highlights from the ORS PSRS 5th International Spine Research Symposium

**DOI:** 10.1002/jsp2.1134

**Published:** 2020-12-02

**Authors:** Lachlan J. Smith, James C. Iatridis, Chitra L. Dahia

**Affiliations:** ^1^ Departments of Neurosurgery and Orthopaedic Surgery Perelman School of Medicine, University of Pennsylvania Philadelphia Pennsylvania USA; ^2^ Translational Musculoskeletal Research Center Corporal Michael J. Crescenz VA Medical Center Philadelphia Pennsylvania USA; ^3^ Leni and Peter W. May Department of Orthopaedics Icahn School of Medicine at Mount Sinai New York New York USA; ^4^ Orthopedic Soft Tissue Research Program Hospital for Special Surgery New York New York USA; ^5^ Department of Cell and Development Biology Weill Cornell Medicine, Graduate School of Medical Sciences New York New York USA

## Abstract

The fifth biennial ORS PSRS International Spine Research Symposium took place from November 3 to 7, 2019, at Skytop Lodge in northeastern Pennsylvania. Organized jointly by the Orthopaedic Research Society and the Philadelphia Spine Research Society, the symposium attracted more than 180 participants from 10 different countries to share the latest advances in basic and preclinical spine research. Following the symposium, participants were invited to submit full‐length manuscripts to this special issue of JOR Spine.

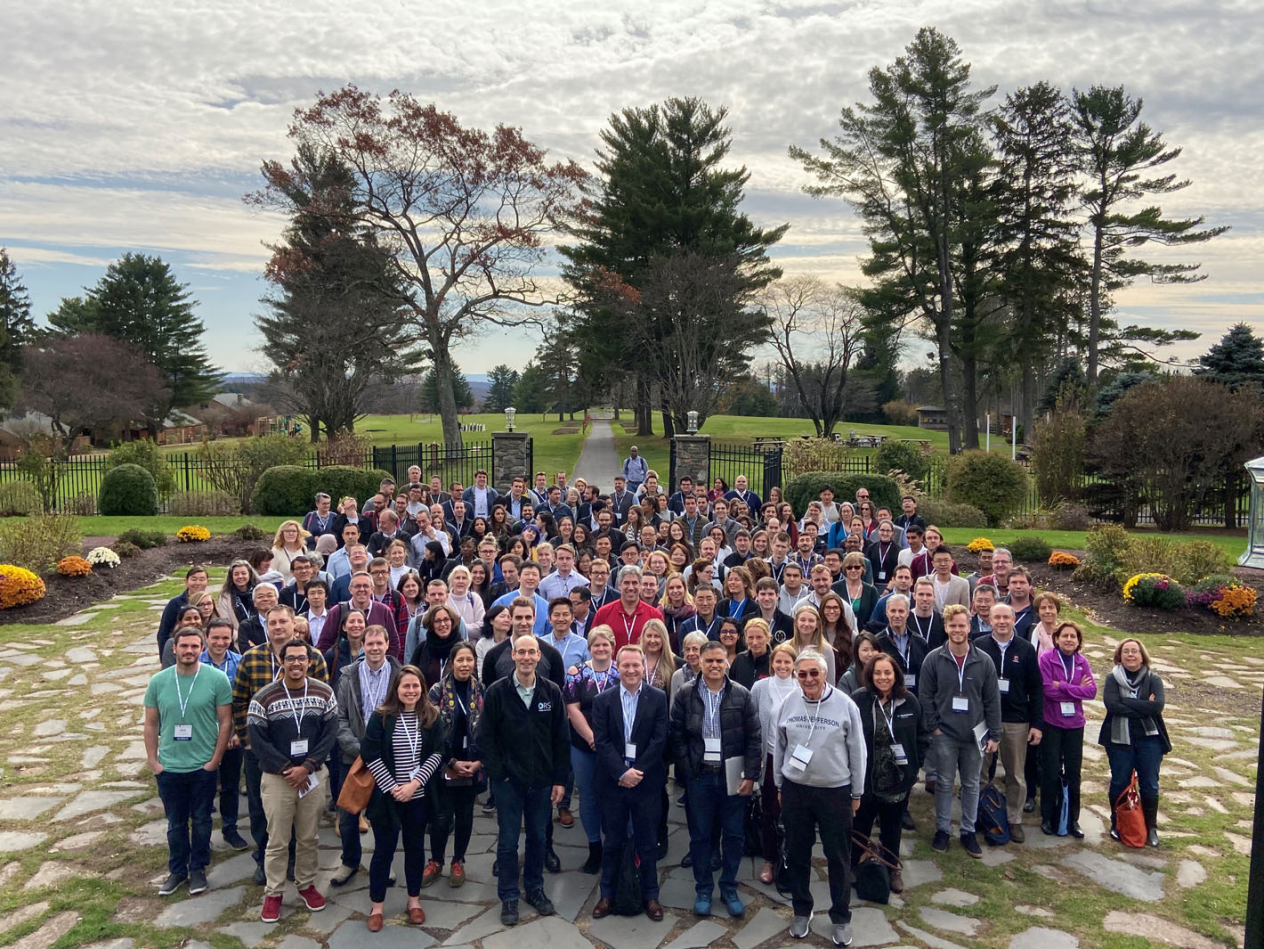

## 1 INTRODUCTION

**FIGURE 1:**
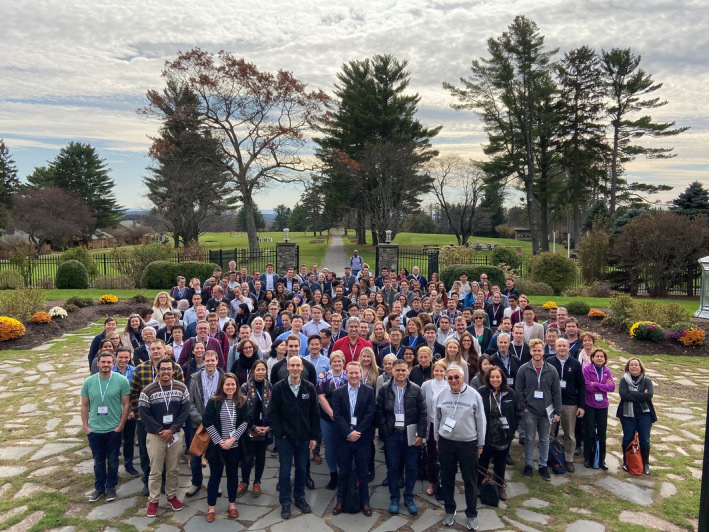
ORS PSRS 5th International Spine Research Symposium attendee group photo

**FIGURE 2:**
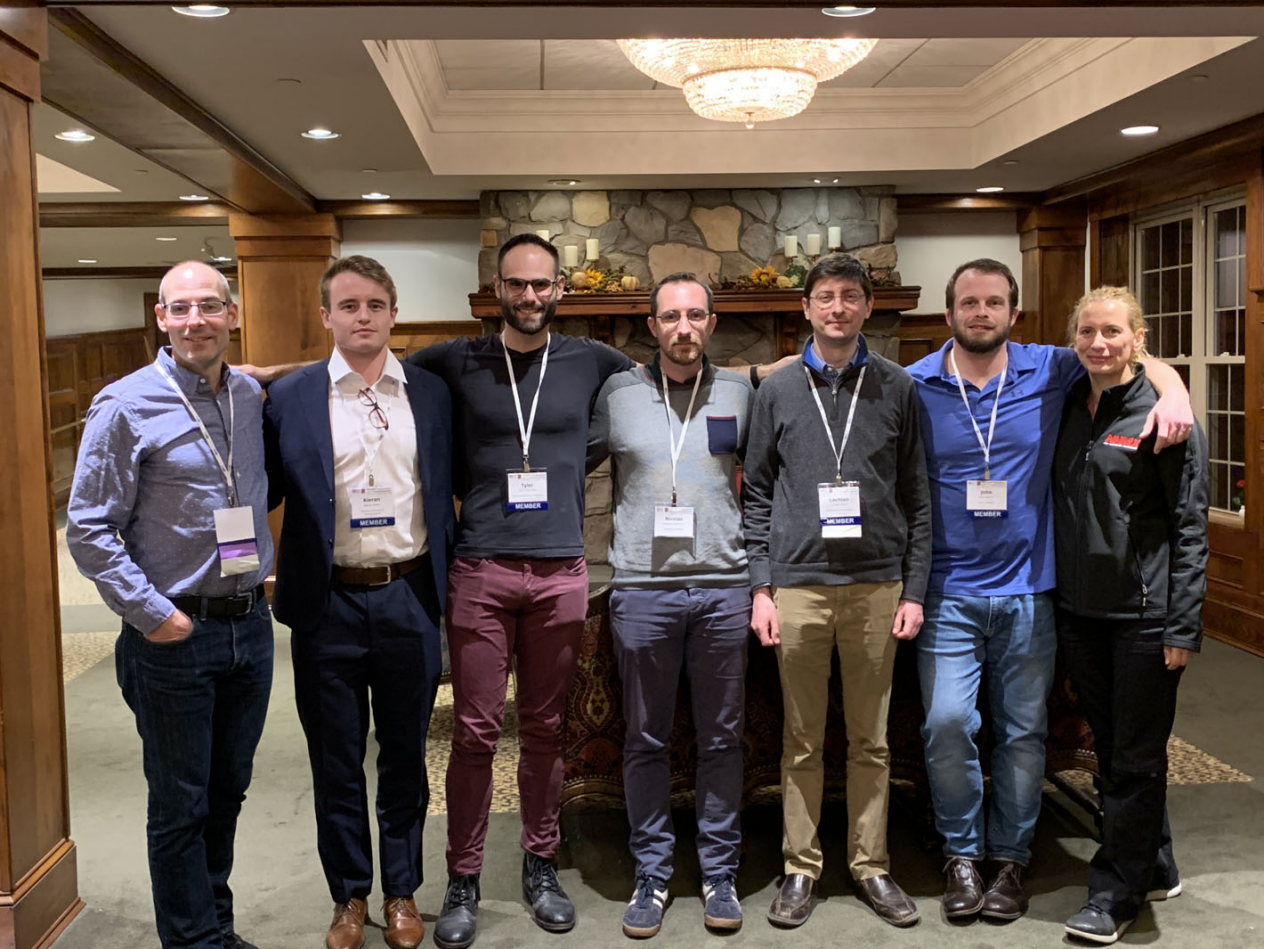
ORS PSRS 5th International Spine Research Symposium trainee podium presentation award winners

**FIGURE 3:**
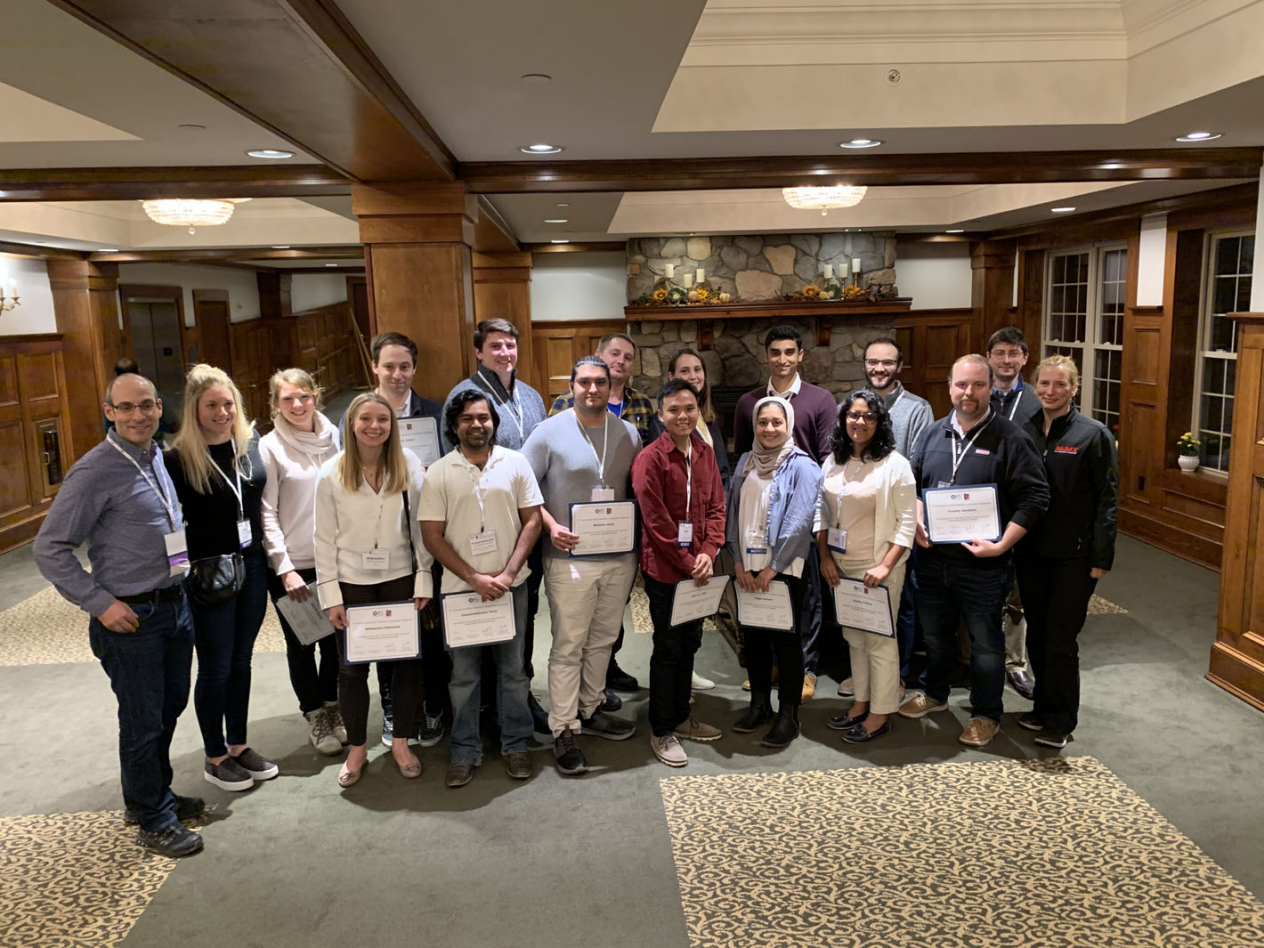
ORS PSRS 5th International Spine Research Symposium trainee poster presentation award winners

The fifth biennial ORS PSRS International Spine Research Symposium took place from November 3 to 7, 2019, at Skytop Lodge in northeastern Pennsylvania (www.ors.org/psrs-2019). Organized jointly by the Orthopedic Research Society and the Philadelphia Spine Research Society, the symposium attracted more than 180 participants from 10 different countries to share the latest advances in basic and preclinical spine research (Figure 1). The three‐and‐a‐half‐day program included 19 invited faculty presentations, and 26 podium and 115 poster presentations predominantly from trainees. Session topics included: Animal Models and Preclinical Studies; Biology, Development, and Pathophysiology; Biomechanics and Tissue Cross‐Talk; Diagnosis, Biomarkers, and Clinical Treatment; Pain Mechanisms; and Next Generation Treatments and Imaging. The 2019 PSRS Lifetime Achievement Award was presented to Dr Michele Battie, for her seminal work on the etiopathogenesis of degenerative and painful conditions of the lumbar spine. Top trainee presentations were recognized by six podium and 12 poster awards sponsored by the PSRS (Figures 2 and 3), and the ORS Spine Section sponsored awards for the two most innovative presentations and two most outstanding early stage investigators. The symposium saw the creation of an expert taskforce to establish uniform guidelines for the histopathological grading of intervertebral disc degeneration. This taskforce was broken into subgroups focused on large, medium, and small animal models, and human tissue, with recommendations from each to be published in an upcoming special issue of this journal. Additionally, a dynamic, interactive workshop on “Behavior and Sensitivity Assays in Preclinical Animal Models of Disc Degeneration and Low Back Pain” was held. Between scientific sessions, participants networked and socialized in the beautiful late fall surroundings of the Pocono Mountains. The symposium concluded with an entertaining, spine‐themed quiz night organized by members of the ORS Spine Section Membership Taskforce. Following the symposium, participants were invited to submit full‐length manuscripts to this special issue of JOR Spine. Highlights are summarized below.

## 2 ISSUE HIGHLIGHTS

### 2.1 Novel imaging and characterization of spine structure, composition, and cellularity

Recent technological advances have enabled spine researchers to characterize healthy and pathological tissue structure, composition, and cellularity with unparalleled sophistication and resolution. Findings not only provide new insights into spine disease pathophysiology, but also establish the target benchmarks for tissue repair and regeneration. Hernandez et al describe a new protocol for 3D imaging of nucleus pulposus (NP) cells in alginate constructs and in their native tissue environment using confocal microscopy, permitting the high‐resolution detection of phenotypical and cytoskeletal changes in these cells without the need for histological processing.^1^ Fearing et al used a pharmacological‐based approach to investigate the relationships between NP cell morphology and gene expression.^2^ They showed that treating degenerate NP cells with verteporfin promoted a rounded, clustered morphology and increased expression of vacuoles and NP‐specific gene markers, and biosynthetic activity. Zeldin et al investigated alterations in the collagen content of human intervertebral discs using second harmonic generation imaging.^3^ They showed that degeneration shifted collagen organization in the AF from a complex to a more uniform structure, and increased fibrotic remodeling in the NP. Sloan et al measured alterations in healthy and experimentally degenerated sheep disc composition using an optimized Fourier transform infrared (FTIR) microscopy technique.^4^ They demonstrated strong correlations between disc collagen and proteoglycan contents, and disc degeneration severity, with superior fidelity compared to traditional histological techniques. Finally, Lakstins et al undertook a detailed cross‐species comparison of the disc cartilage end plate.^5^ They found significant differences in the structure, composition, cellularity and gene expression of canine and bovine end plates compared to those from human discs, to help inform appropriate animal model selection and improved clinical relevance.

### 2.2 Disc degeneration and low back pain in the context of aging and comorbidities

Aging and comorbidities such as diabetes are significant risk factors for developing symptomatic intervertebral disc degeneration. Kritschil et al investigated the role of insulin‐like growth factor I (IGF‐I) signaling on the progression of disc degeneration in aging mice.^6^ Interestingly, they showed that diminished IGF‐I bioavailability confers both the beneficial effects of decreased disc cell senescence and extracellular matrix catabolism, whilst at the same time negatively impacting proteoglycan production. Hoy et al used second harmonic generation and collagen‐hybridizing peptide imaging to assess compositional changes in mouse intervertebral discs associated with high dietary intake of advanced glycation end‐products (AGEs).^7^ Their results showed receptor‐dependent collagen disruption associated with AGE accumulation at multiple hierarchical levels. Finally, Vincent et al examined adaptions in the somatosensory nervous system in aging mice, demonstrating that the dorsal root ganglia exhibit inflammatory molecular and cellular changes that are associated with increased peripheral sensitization.^8^

### 2.3 Clinically‐relevant animal models of spinal disorders

Appropriate selection and implementation of clinically‐relevant large animal models are critical steps for the successful translation of new spinal therapeutics. Lee et al provide a timely review of canine models of spinal disorders, highlighting the applicability of these models for advancing translational research, including diagnosis, prognosis, prevention, and treatment of disc degeneration and low back pain.^9^

## CONFLICT OF INTEREST

The authors have no conflicts of interest to disclose.
